# Versatile Use of the Free Anterolateral Thigh Flap in Pediatric Reconstruction: A Report of Two Cases

**DOI:** 10.7759/cureus.110911

**Published:** 2026-06-15

**Authors:** Mohammed Benlili, Doha Arreyouchi, Ayat Allah Oufkir

**Affiliations:** 1 Plastic and Reconstructive Surgery, Mohammed First University, Oujda, MAR

**Keywords:** anterolateral thigh flap, facial reconstruction, free flap, microsurgery, pediatric reconstruction, xeroderma pigmentosum

## Abstract

Complex soft tissue defects in children require reconstructive solutions that are stable, adaptable, and compatible with long-term growth. The free anterolateral thigh flap offers several advantages in this setting because it can be tailored according to the defect, either as a durable resurfacing flap or as a source of vascularized tissue volume. We report two pediatric cases managed with a free anterolateral thigh flap for different reconstructive purposes. The first patient was an eight-year-old boy with a painful and unstable post-traumatic scar of the right heel after a road traffic accident and previous split-thickness skin grafting. After complete scar excision, reconstruction was performed using an ipsilateral fasciocutaneous anterolateral thigh flap. At two-year follow-up, the flap remained stable, painless, and free of ulceration; the visual analog scale pain score decreased from 6/10 preoperatively to 0/10, and the patient resumed school activities, walked independently with definitive adapted footwear, and required no secondary debulking procedure. The second patient was a seven-year-old boy with xeroderma pigmentosum who presented with a large nasal squamous cell carcinoma extending to the upper lip and pre-nasal cheek region. After tumor excision with 1 cm lateral margins, resection through a healthy deep plane, and bilateral cervical lymph node dissection, reconstruction was achieved using a free fasciocutaneous anterolateral thigh flap harvested from the left thigh. The cervical lymph node dissection was negative, and no adjuvant radiotherapy was administered after multidisciplinary tumor board discussion because complete excision had been achieved and the benefit-risk balance was considered unfavorable in the context of xeroderma pigmentosum. At one-year clinical follow-up, there was no evidence of recurrence. These two cases illustrate how the free anterolateral thigh flap may address different pediatric reconstructive needs, ranging from durable heel coverage to vascularized filling of a central facial cavity after oncologic resection.

## Introduction

Reconstruction of soft tissue defects in children requires a strategy that goes beyond simple wound closure. The reconstructed area must tolerate growth, preserve function, avoid recurrent breakdown, and minimize donor-site consequences. These issues become particularly important when the defect involves a functional weight-bearing region, such as the heel, or an exposed aesthetic and functional unit, such as the central face.

The heel is a difficult site to reconstruct because it is exposed to repeated pressure and shear forces during walking. A fragile grafted or scarred surface may become painful, unstable, or ulcerated, even in the absence of an exposed bone. In contrast, reconstruction of the central face often requires vascularized tissue capable of filling a deep defect, restoring facial contour, and supporting functional recovery after tumor resection.

The anterolateral thigh flap has become a major option in microsurgical reconstruction since its original description by Song et al [1]. Its popularity is related to its long vascular pedicle, large skin paddle, and ability to be harvested with different tissue components. Depending on the clinical situation, it may be used as a thin fasciocutaneous flap or as a bulkier flap for volume restoration [2,3].

In children, the use of free flaps may be technically more demanding because of smaller vessels, vasospasm, and the need for careful postoperative monitoring. Nevertheless, pediatric studies have shown that the anterolateral thigh flap can be used safely when appropriate planning and microsurgical expertise are available [4,5].

The primary objective of this report is to illustrate the versatility of the free anterolateral thigh flap in two distinct pediatric reconstructive settings: durable weight-bearing heel coverage after post-traumatic scarring and vascularized volumetric reconstruction after extensive oncologic resection of the central face. By presenting these two contrasting cases, we aim to demonstrate how the same microsurgical option can be adapted to different anatomical sites, functional requirements, and reconstructive goals in children.

## Case presentation

Case 1

An eight-year-old boy was admitted for treatment of an unstable scar of the right heel. His history dated back to the age of four years, when he was struck by a car as a pedestrian. The accident caused severe soft tissue damage to the right heel, which was initially managed with split-thickness skin grafting.

**Figure 1:**
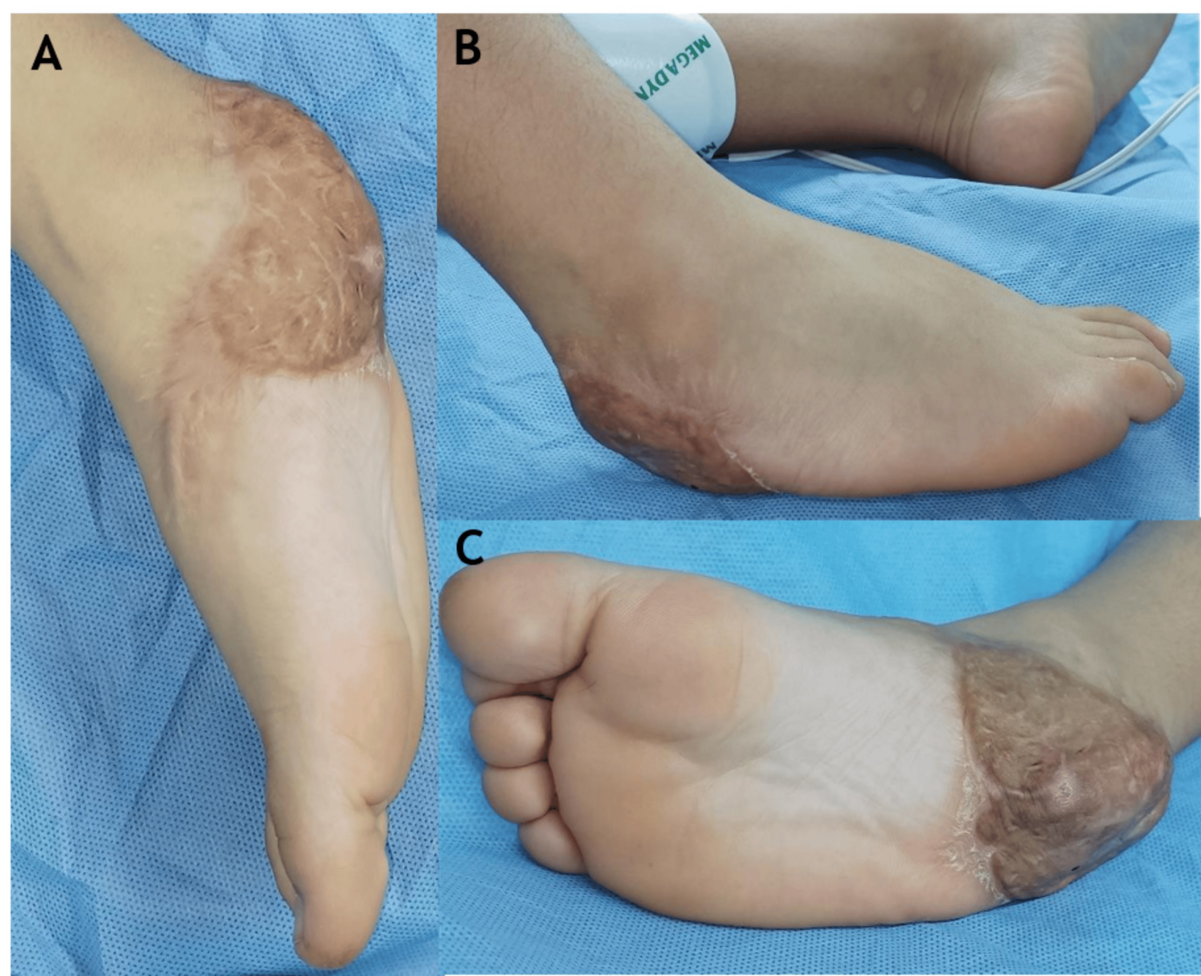
Preoperative clinical appearance of the unstable post-traumatic scar of the right heel. (A) Medial view of the right heel showing the unstable scar. (B) Lateral view demonstrating the extension of the scar over the heel region. (C) Plantar view showing the involvement of the weight-bearing area of the heel. Written informed consent was obtained from the parents/legal guardians of the patient for the publication of this case report and the accompanying clinical images.

At presentation, clinical examination showed a 9 × 8 cm atrophic scar involving the posterior and plantar aspects of the right heel. The scar was adherent to the calcaneus, painful, and unstable, causing pain during weight-bearing and difficulty walking. There was no clinical exposure of the calcaneus at initial examination. The patient reported pain rated at 6/10 on the visual analog scale. No evidence of osteitis was found during the preoperative assessment, and the vascular status of the lower limb was preserved (Figure 1). No fixed equinus deformity or clinically significant Achilles tendon shortening was identified during the preoperative assessment. Therefore, no tendon lengthening procedure was indicated.

**Figure 2:**
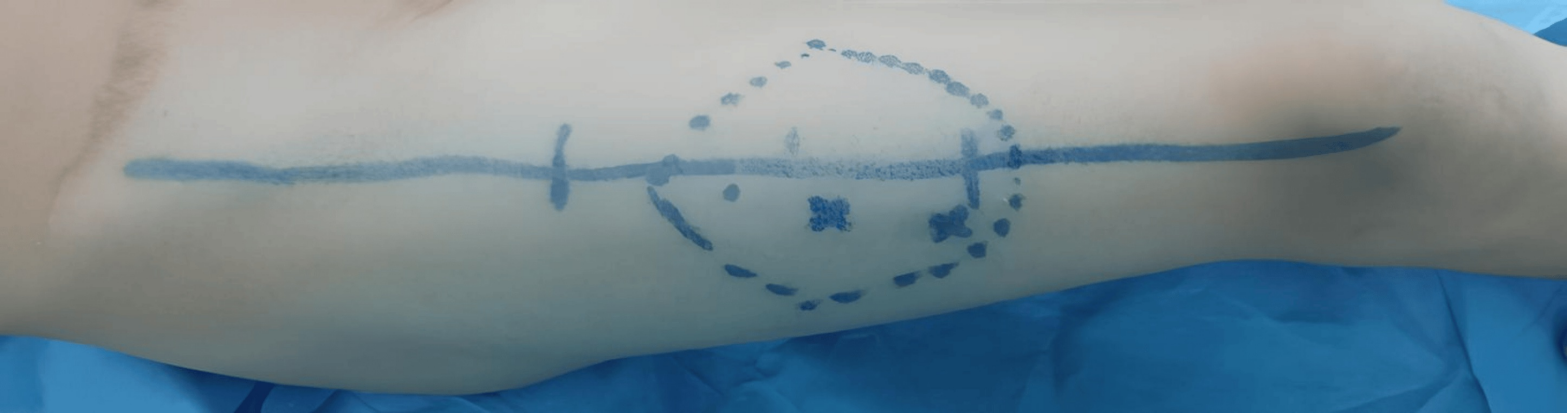
Preoperative marking of the anterolateral thigh flap. Preoperative design of the ipsilateral fasciocutaneous anterolateral thigh flap measuring 10 × 9 cm. The flap was planned along the long axis of the thigh, and the perforators identified by preoperative ultrasound mapping were marked within the skin paddle. Written informed consent was obtained from the parents/legal guardians of the patient for the publication of this case report and the accompanying clinical images.

Preoperative workup included Doppler ultrasonography of the dorsalis pedis vessels and ultrasound mapping of the perforators of the anterolateral thigh flap. The flap was designed on the ipsilateral thigh according to the long axis of the thigh, and the perforators identified by ultrasound were marked within the planned skin paddle (Figure 2). Because the lesion involved a weight-bearing area, with an unstable adherent scar and insufficient local tissue for reliable reconstruction, a free anterolateral thigh flap was selected.

**Figure 3:**
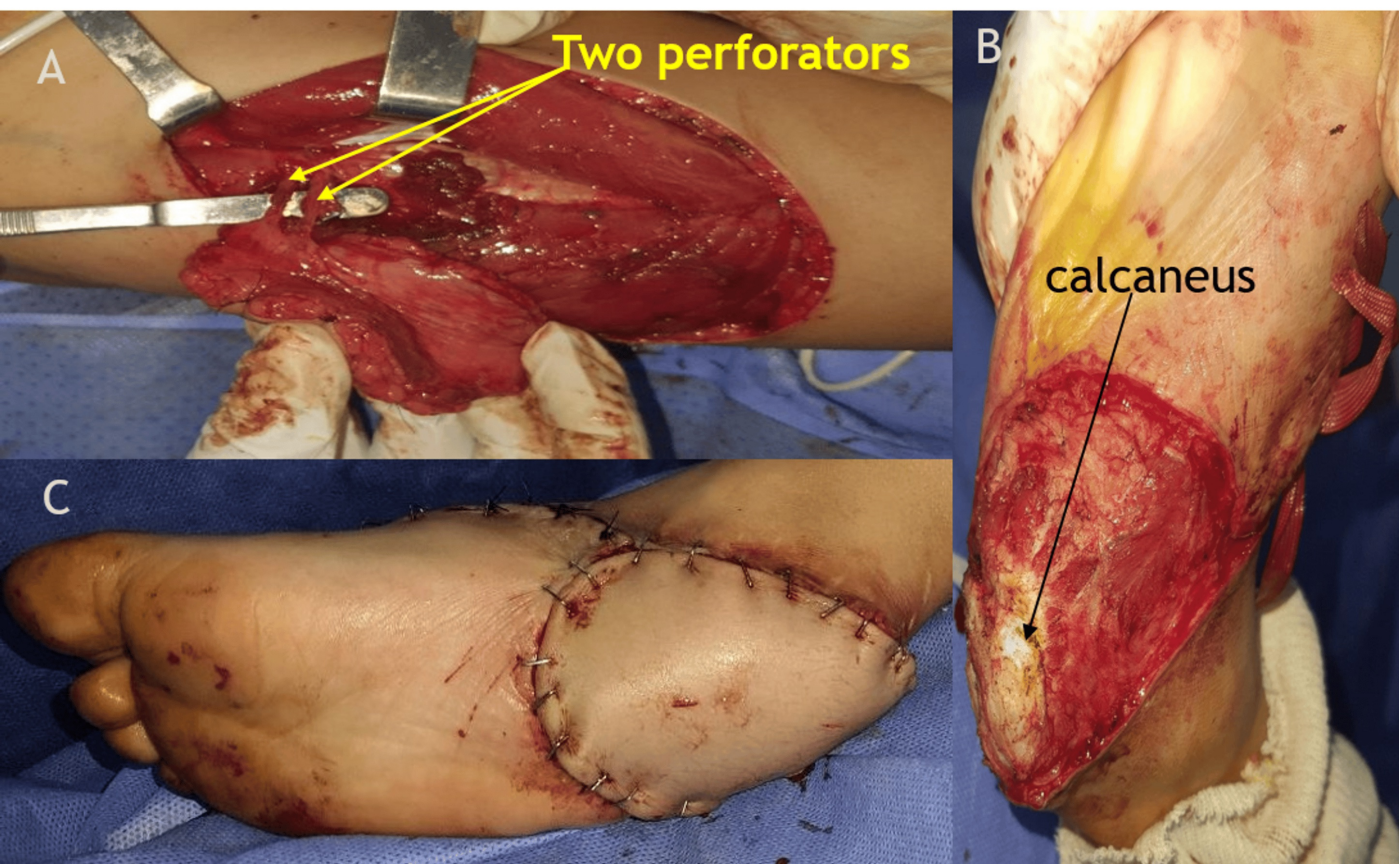
Intraoperative findings and flap inset during right heel reconstruction. (A) Intraoperative elevation of the ipsilateral fasciocutaneous anterolateral thigh flap based on two perforators; the yellow arrows indicate the two perforator vessels supplying the flap. (B) Heel defect after complete scar excision and debridement, showing exposure of the calcaneus; the black arrow indicates the exposed calcaneal bone. (C) Appearance after end-to-end microvascular anastomosis and flap inset over the heel defect. Written informed consent was obtained from the parents/legal guardians of the patient for the publication of this case report and the accompanying clinical images.

Surgery was performed under general anesthesia. The scar tissue was completely excised, and after debridement, the calcaneus was exposed intraoperatively. Reconstruction was performed using an ipsilateral fasciocutaneous anterolateral thigh flap measuring 10 × 9 cm along its long axis. The flap was harvested as a non-sensate flap, and no nerve coaptation was performed. The flap was elevated on two perforators (Figure 3A). After complete excision of the scar tissue and preparation of the recipient site, the heel defect was covered with the free flap (Figure 3B). Although two veins were available in the flap, only one venous anastomosis was performed. The arterial anastomosis was performed to the dorsalis pedis artery in an end-to-end fashion, and the venous anastomosis was also performed end-to-end. The ischemia time was 40 minutes, and the total operative time was four hours. The donor site was covered with a split-thickness skin graft. Final flap inset was performed after microvascular anastomosis and fixation to the heel defect (Figure 3C).

The department protocol for free flap anticoagulation was followed. It consisted of an intravenous heparin bolus of 30-50 IU/kg before clamping, followed by prophylactic low-molecular-weight heparin administered subcutaneously for five days. Flap monitoring was performed at one, two, four, six, and 12 hours after surgery, followed by regular close monitoring according to the department protocol.

The postoperative course was uncomplicated. There was no venous congestion, arterial insufficiency, infection, or flap necrosis. Complete wound healing was obtained by postoperative day 15. Partial weight-bearing was started one month after surgery. The patient initially walked with crutches and then progressively without assistance. Adapted footwear was prescribed according to the shape of the reconstructed heel.

**Figure 4:**
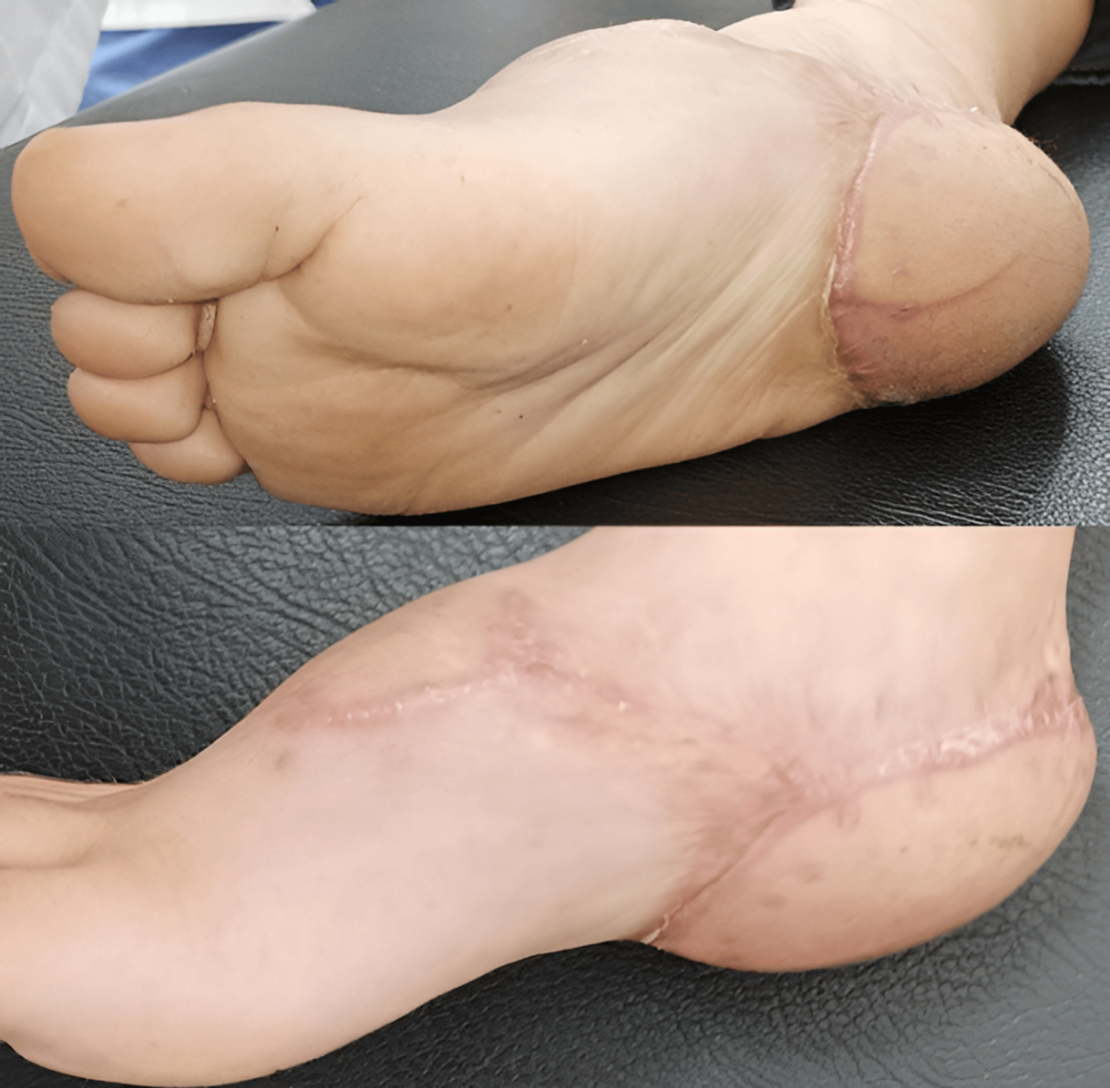
Two-year postoperative outcome after right heel reconstruction. Clinical images showing stable coverage of the right heel two years after reconstruction with a free anterolateral thigh flap. Written informed consent was obtained from the parents/legal guardians of the patient for the publication of this case report and the accompanying clinical images.

At two-year follow-up, the reconstruction remained stable. The patient had painless weight-bearing, no ulceration, and no recurrent wound breakdown. Pain resolved completely, with a postoperative visual analog scale pain score of 0/10. He was able to walk independently, resumed school activities, and used definitive adapted footwear. No secondary debulking or other revision procedure was required (Figure 4).

Case 2

**Figure 5:**
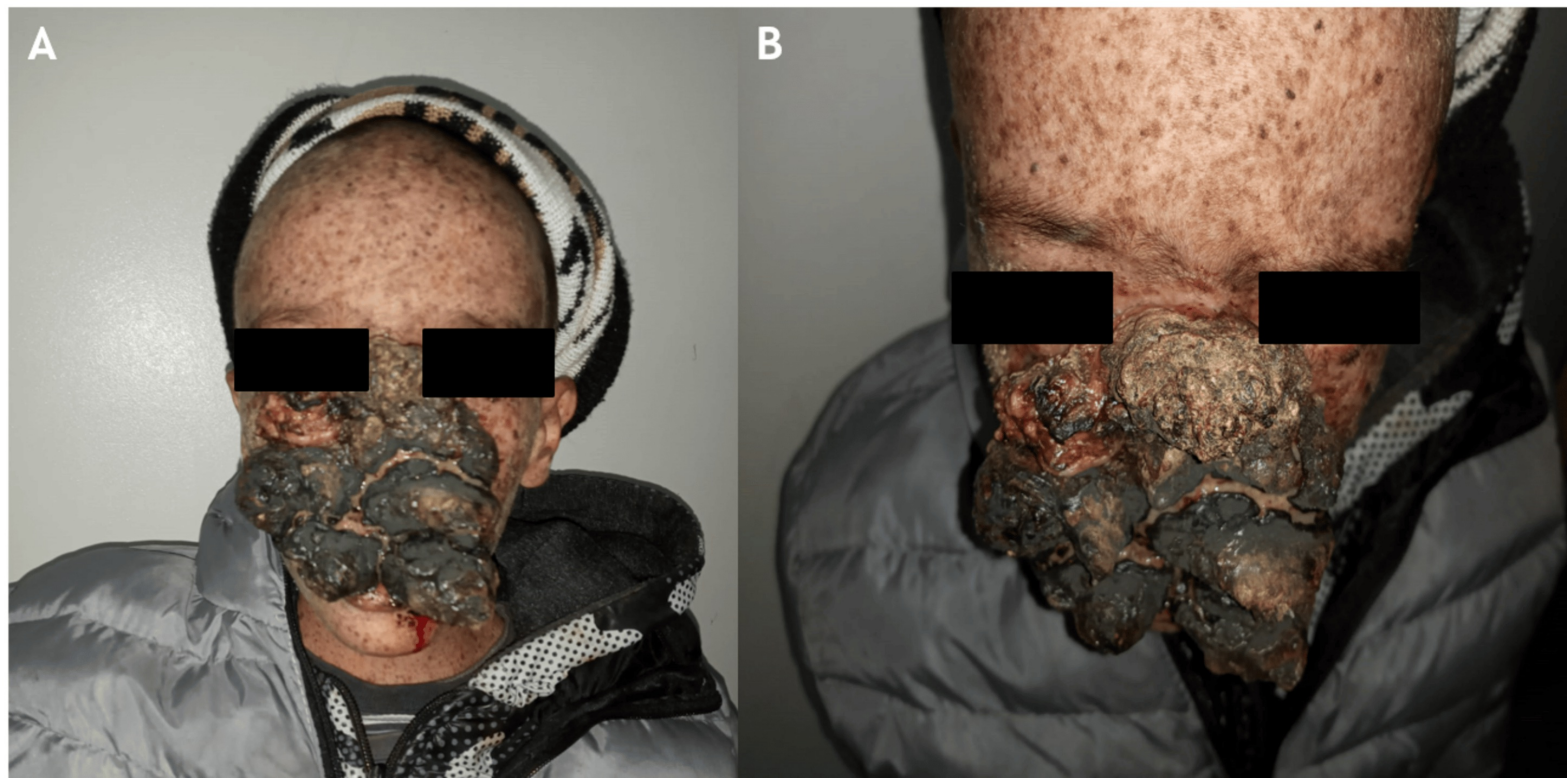
Preoperative images of the seven-year-old boy with xeroderma pigmentosum showing a large ulceronecrotic nasal tumor extending to the upper lip and the pre-nasal cheek region. (A) Frontal view demonstrating the overall extent of the ulceronecrotic nasal tumor and midfacial involvement. (B) Close-up view highlighting the tumor extension to the upper lip and pre-nasal cheek region. Written informed consent was obtained from the parents/legal guardians of the patient for the publication of this case report and the accompanying clinical images.

A seven-year-old boy with xeroderma pigmentosum was admitted for management of an advanced tumor of the central face. Clinical examination revealed a large ulceronecrotic nasal lesion extending to the upper lip and the pre-nasal cheek region. The tumor measured approximately 13 × 9 cm. It caused major functional impairment, including complete nasal obstruction with inability to breathe through the nose and limitation of oral feeding because of involvement of the central facial region (Figure 5).

A preoperative biopsy was performed and confirmed a moderately differentiated squamous cell carcinoma. Preoperative imaging included cervicofacial and thoracoabdominopelvic computed tomography. The cervicofacial scan showed a locally advanced nasal tumor with destruction of nasal bones, the ascending processes of the maxillae, and the anterior walls of both maxillary sinuses. Cervical lymphadenopathy was suspected on imaging. No distant metastasis was identified.

**Figure 6:**
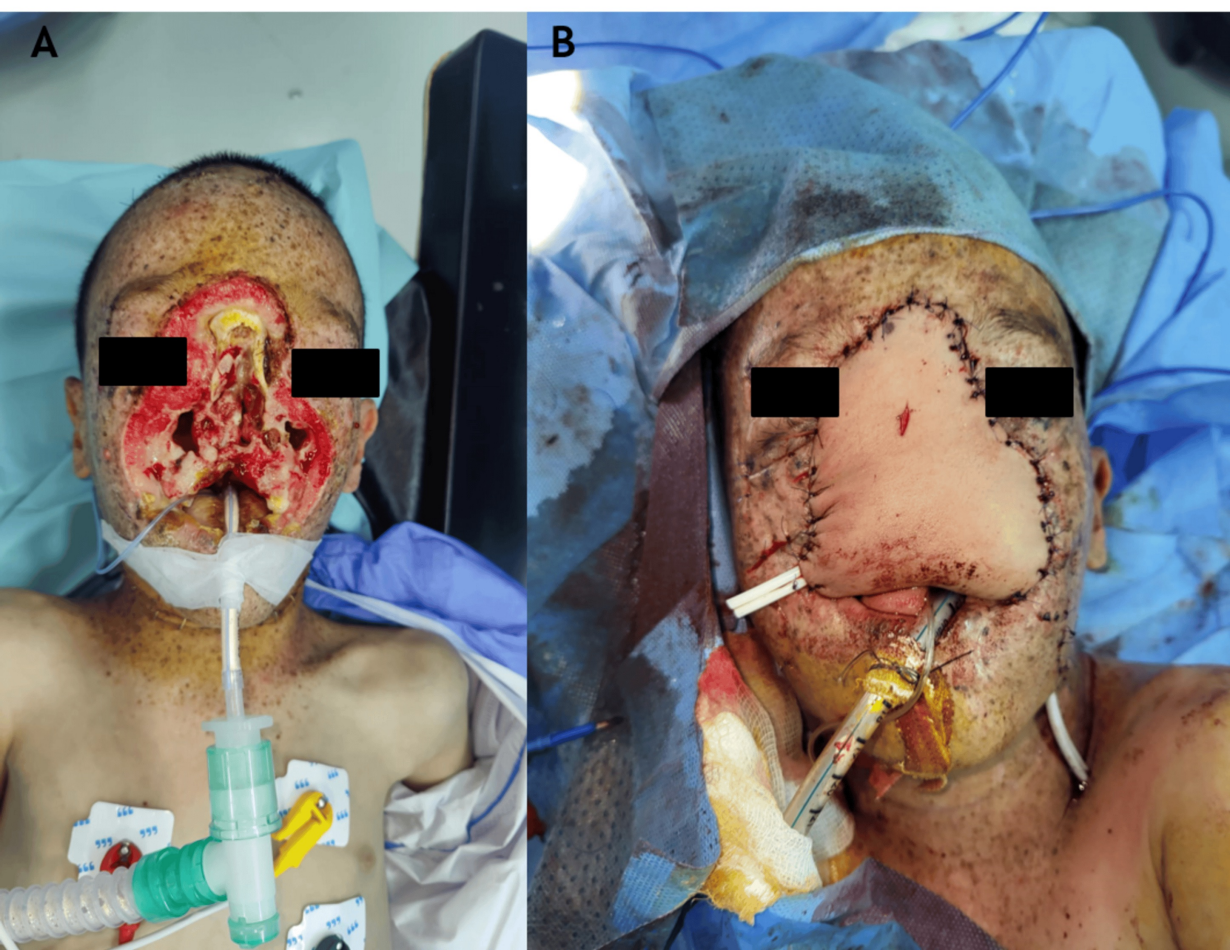
Intraoperative stages of reconstruction after excision of the central facial tumor. (A) Intraoperative view after oncologic resection, demonstrating the central facial defect created after excision of the nasal squamous cell carcinoma. (B) Final inset of the free fasciocutaneous anterolateral thigh flap after microvascular anastomosis and fixation of the flap to the recipient site. Written informed consent was obtained from the parents/legal guardians of the patient for the publication of this case report and the accompanying clinical images.

The patient underwent tumor excision and bilateral cervical lymph node dissection under general anesthesia. The oncologic part of the procedure, including tumor resection and lymph node dissection, lasted five hours. Tumor excision was performed with 1 cm lateral margins and a resection plane through macroscopically healthy deep tissue. The resection resulted in a large central facial defect (Figure 6A). The defect involved the external nose, with loss of the nasal soft tissues and underlying bony framework, including the nasal bones, the ascending processes of the maxillae, and the anterior walls of both maxillary sinuses. No immediate cartilaginous reconstruction was performed. Final histopathological examination confirmed complete excision with free margins, including a healthy deep plane. The cervical lymph node dissection was negative, with no metastatic lymph nodes identified among 22 removed nodes.

After confirmation of oncologic resection, reconstruction was performed using a free anterolateral thigh flap. The primary reconstructive objective was to provide well-vascularized tissue with adequate volume to fill the residual central facial cavity and restore facial contour. Immediate nasal airway reconstruction was not the primary objective at this stage. Creation of the nasal alae was therefore deferred, and no nasal stents were used during the initial reconstructive procedure. The flap was not prelaminated, and no internal nasal lining was reconstructed.

A free fasciocutaneous anterolateral thigh flap was harvested from the left thigh under the same general anesthesia. Two perforators were identified. The flap was anastomosed to the facial vessels using end-to-end arterial and venous anastomoses. The ischemia time was 45 minutes. The reconstructive stage lasted three hours. No additional flap remodeling was required. The donor site was covered with a split-thickness skin graft, and complete graft take was obtained. The flap was then inset into the central facial defect after microvascular anastomosis and fixation to the recipient site (Figure 6B).

The postoperative course was favorable, with no vascular compromise, flap necrosis, infection, or donor-site complication. Oral feeding was restored after reconstruction, and the patient was able to breathe comfortably through the mouth. The indication for adjuvant radiotherapy was discussed during a multidisciplinary tumor board meeting. In view of the patient's xeroderma pigmentosum, complete surgical excision, absence of lymph node metastasis, and the expected long-term morbidity of radiotherapy, the benefit-risk balance was considered unfavorable, and the decision was made not to administer adjuvant radiotherapy.

**Figure 7:**
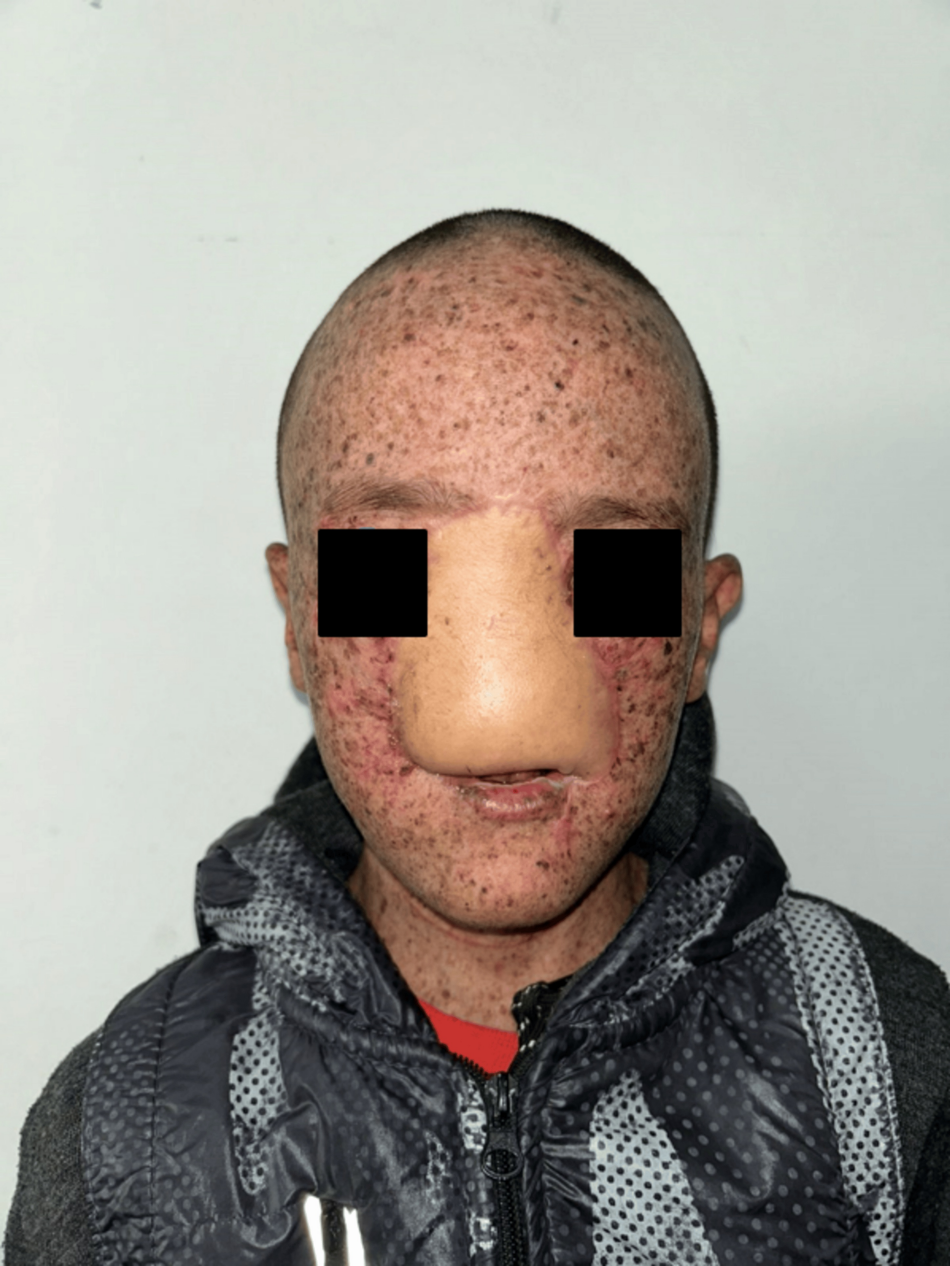
One-year follow-up after reconstruction of the central facial defect. Written informed consent was obtained from the parents/legal guardians of the patient for the publication of this case report and the accompanying clinical images.

Follow-up was based on regular clinical examination for one year. At one-year follow-up, the flap was well integrated, with stable soft tissue coverage and restoration of central facial volume. There was no clinical evidence of local recurrence or distant disease (Figure 7).

The possibility of secondary nasal reconstruction, including dorsal augmentation to improve nasal projection, was discussed with the patient and his family. However, at the time of writing, they were not in favor of additional surgical procedures. Further refinements may be reconsidered in the future according to the patient's functional and aesthetic needs and the family's preferences.

## Discussion

The two cases presented in this report illustrate two different problems in pediatric reconstruction. The first was a functional problem involving a weight-bearing heel scar. The second was a complex oncologic defect of the central face in a child with xeroderma pigmentosum. Although the anatomical sites and reconstructive goals were different, both situations were managed with the same flap: the free anterolateral thigh flap.

In children, the choice of reconstruction must take into account both the immediate defect and the long-term consequences. A reconstruction that is acceptable in the early postoperative period may later become problematic if it is unstable, bulky, painful, or poorly adapted to growth. This is particularly relevant in the foot, where repeated mechanical stress can lead to ulceration, and in the face, where even minor contour irregularities may have important functional and psychosocial consequences.

The anterolateral thigh flap is well suited to this type of reconstructive thinking because it is not limited to one specific use. It may be harvested with skin, fascia, subcutaneous fat, or muscle, and its thickness can be adapted to the recipient site. Since its first description, it has become one of the most frequently used perforator flaps in microsurgery [1]. Its versatility has been particularly emphasized in head and neck reconstruction, where it can be adapted to cutaneous, mucosal, and volumetric defects [2,3].

Pediatric microsurgery has specific technical constraints. Smaller vessels, vasospasm, and the need for precise postoperative monitoring may increase the complexity of free tissue transfer. However, the literature supports the reliability of the anterolateral thigh flap in children. Gharb et al. reported a pediatric series of free anterolateral thigh flaps with no complete flap loss, showing that this flap can be used successfully in different anatomical regions in children [4]. Other pediatric series have also supported its role in extremity reconstruction [5].

In our first case, the indication was not an acute open defect but a chronic unstable post-traumatic scar of the heel. The patient had previously undergone split-thickness skin grafting after a road traffic accident. Although the graft provided coverage, the resulting scar was atrophic, adherent, painful, and functionally disabling. This situation highlights an important point: in the heel, coverage alone is not always sufficient. The reconstructed surface must be stable enough to tolerate walking.

The plantar heel is a specialized structure. It is exposed to pressure, friction, and shear forces during standing and ambulation. A fragile scar or grafted surface may cause pain, limit gait, and predispose to recurrent breakdown. Several reconstructive options have been described for heel and foot defects, including local flaps, regional flaps, medial plantar flaps, sural flaps, and free flaps. However, when local tissues are scarred or insufficient, free tissue transfer may provide a more reliable solution.

In pediatric foot and ankle reconstruction, the free anterolateral thigh flap has been reported as a useful option for complex soft tissue defects [5]. Bekara et al. also discussed the role of free anterolateral thigh flaps compared with pedicled sural flaps in pediatric dorsal foot and ankle reconstruction, showing that both options may be useful depending on the defect and local conditions [6]. In our patient, the main goal was to replace an unstable adherent scar with vascularized soft tissue that could tolerate weight-bearing. At two-year follow-up, the flap remained stable and painless, with no ulceration, no need for secondary debulking, and complete pain relief, as shown by a postoperative visual analog scale pain score of 0/10. No clinically significant flap wobbling or instability was observed during follow-up, and adapted footwear provided satisfactory functional support. The child resumed school activities and walked independently with adapted footwear. This favorable evolution supports the use of the anterolateral thigh flap in selected pediatric heel reconstructions, including secondary reconstruction of unstable scars.

The second case demonstrates another use of the flap: reconstruction after oncologic resection of a central facial tumor. Xeroderma pigmentosum is a rare inherited disorder characterized by defective repair of ultraviolet-induced DNA damage. Patients are at high risk of developing cutaneous malignancies at an early age, particularly in sun-exposed areas such as the face [7,8]. Squamous cell carcinoma in children with xeroderma pigmentosum can be locally aggressive and may require wide resection.

Our patient presented with a large nasal squamous cell carcinoma extending to the upper lip and pre-nasal cheek region. Imaging showed extensive local destruction, and cervical lymphadenopathy was suspected radiologically. However, final histopathological analysis after bilateral cervical lymph node dissection found no metastatic lymph nodes among 22 removed nodes. This distinction is clinically important because radiological suspicion does not always correspond to pathological nodal disease.

The reconstructive challenge after tumor excision was to fill the residual central facial cavity with vascularized tissue and restore a more acceptable facial contour. The objective was therefore primarily volumetric and protective rather than immediate nasal airway restoration. In this setting, the anterolateral thigh flap provided sufficient tissue volume and a reliable vascular pedicle. The flap was anastomosed to the facial vessels, and no additional remodeling was required. The postoperative course was favorable, with restoration of oral feeding and comfortable mouth breathing.

The decision not to administer adjuvant radiotherapy was made after multidisciplinary tumor board discussion. It was based on complete surgical excision with 1 cm lateral margins and a healthy deep plane, negative lymph node dissection, absence of distant metastasis, and the specific context of xeroderma pigmentosum. Because of the expected long-term morbidity of radiotherapy in this child, the benefit-risk balance was considered unfavorable. In our report, the therapeutic strategy relied on complete surgical excision, histological control, vascularized reconstruction, and close clinical surveillance over one year.

Several technical aspects contributed to the successful outcomes in these two cases. Preoperative vascular assessment was performed in the heel case, including Doppler evaluation of the dorsalis pedis vessels and perforator mapping. In both cases, two perforators were identified. Recipient vessels were chosen according to the defect: the dorsalis pedis vessels for the heel reconstruction and the facial vessels for the central facial reconstruction. Both procedures were performed under general anesthesia, and both donor sites were managed with split-thickness skin grafting.

Postoperative monitoring was also essential. In the first case, flap viability was assessed at one, two, four, six, and 12 hours after surgery, followed by close monitoring according to the department protocol. No vascular compromise occurred in either patient. In pediatric free flap surgery, early recognition of venous congestion or arterial insufficiency remains crucial because timely revision may determine flap survival.

This report has limitations. It includes only two patients, and the two defects were different in location, etiology, and reconstructive goal. Therefore, no general conclusion can be drawn regarding superiority over other reconstructive options. Functional assessment was also mainly clinical. In the heel case, no formal gait analysis was performed, although the patient achieved stable and painless walking at two years, with a postoperative visual analog scale pain score of 0/10, return to school activities, and no need for secondary debulking. In the facial case, aesthetic and psychosocial outcomes were not evaluated using standardized instruments. Longer follow-up is required because children with xeroderma pigmentosum remain at risk of local recurrence and new cutaneous malignancies.

Despite these limitations, the two observations underline the adaptability of the anterolateral thigh flap in pediatric reconstruction. In one child, it restored a stable weight-bearing surface. In the other, it provided vascularized tissue for central facial reconstruction after oncologic resection. These two different applications support the role of the free anterolateral thigh flap as a valuable option in selected complex pediatric defects.

## Conclusions

The free anterolateral thigh flap is a useful and adaptable option for complex pediatric reconstruction. In our first case, it provided durable coverage of an unstable post-traumatic heel scar, with stable painless weight-bearing, a postoperative visual analog scale pain score of 0/10, return to school activities, no ulceration, and no need for secondary debulking at two-year follow-up. In our second case, it allowed vascularized filling of a central facial cavity after complete resection of squamous cell carcinoma in a child with xeroderma pigmentosum, with no clinical recurrence at one-year follow-up. Careful patient selection, preoperative planning, meticulous microsurgical technique, multidisciplinary decision-making, and close postoperative monitoring are essential. Long-term follow-up remains necessary to evaluate growth-related changes, functional durability, donor-site morbidity, and oncologic outcomes.
